# Greater smoking intensity may be linked to early smoking initiation among Filipinos: Evidence from the 2021 Global Adult Tobacco Survey Philippines

**DOI:** 10.18332/tpc/194485

**Published:** 2024-11-13

**Authors:** Samuel Brando H. Piamonte

**Affiliations:** 1 Department of Behavioral Sciences, University of the Philippines Manila, Manila, Philippines

**Keywords:** smoking, smoking initiation, smoking intensity, Global Adult Tobacco Survey, Philippines

## Abstract

**INTRODUCTION:**

Understanding the relationship between age at smoking initiation and later smoking intensity is crucial for assessing future health consequences of smoking early and informing strategies to prevent and reduce tobacco use. This study explores the relationship between the two smoking-related behaviors among Filipino daily smokers.

**METHODS:**

Secondary data analyses from the 2021 Global Adult Tobacco Survey Philippines were performed. This study covers those who were reported to be daily smokers. The outcome of interest was smoking intensity, measured as the number of manufactured cigarettes consumed per day, while the main predictor was age at smoking initiation. Data from 2260 participants were analyzed. Negative binomial regression was used to test for the relationship between age at smoking initiation and smoking intensity while controlling for sociodemographic variables and other smoking-related behaviors.

**RESULTS:**

The average age at which daily smoking commenced was 20.93 (SD=6.35) years, while the average number of manufactured cigarettes consumed per day was 9.50 (SD=7.26). Age at smoking initiation was a significant predictor of smoking intensity, even after controlling for potential confounders. Each additional year in the age at which smoking was initiated was associated with a 1.55% decrease in smoking intensity in the adjusted model (β= -0.0155, p<0.0001). Other predictors of smoking intensity were current age (β=0.0072; 95% CI: 0.0050–0.0094, p<0.0001), sex (β= -0.1146; 95% CI: -0.2157 – -0.0136, p=0.0262), and smoking rules at home (β=0.1807; 95% CI: 0.1175–0.2439, p<0.0001).

**CONCLUSIONS:**

Greater smoking intensity may be linked to early smoking initiation among Filipino adult daily smokers. The results may support interventions that target younger ages to curb heavy tobacco use at later ages.

## INTRODUCTION

Smoking is a leading cause of preventable death and disease worldwide, where 1 in 10 deaths globally is caused by tobacco use^1^. In the South-East Asian region, it is estimated that around 4 million all-cause deaths and 105000 lung cancer deaths annually, are attributable to tobacco^2^. In response to this global crisis, the World Health Organization introduced the Framework Convention on Tobacco Control and the MPOWER strategy, which aim to reduce tobacco demand and supply through a set of evidence-informed measures. However, smoking continues to pose a significant public health challenge despite global efforts to reduce tobacco use.

With the significant impact of smoking, it is critical to emphasize factors that contribute to smoking behavior. One key area of focus is smoking initiation, particularly the age at which individuals begin smoking, as this has been illustrated to influence long-term health outcomes and smoking intensity^3^. However, reducing initiation continues to be a challenge. Hence, the importance of strengthening efforts to prevent early smoking initiation, particularly among younger populations, should be emphasized in health policy^4^.

Previous studies on smoking initiation have identified numerous health and behavioral problems associated with early onset of smoking. Starting to smoke at a younger age is linked to a higher risk of developing lung cancer^5^ and increased risks for cardiovascular/metabolic and pulmonary diseases as well as other smoking-related cancers^6^. Additionally, early smoking initiation is associated with higher odds of nicotine dependence and lower odds of successfully quitting smoking^7^. Individuals who begin smoking at a younger age are also more likely to struggle with maintaining continuous smoking abstinence^8^.

Furthermore, evidence suggests that smoking initiation at younger ages is a predictor of heavier smoking intensity later in life. This relationship has been observed across various populations and groups, including those in Catalonia, Spain^9^, Northern Italy^10^, adult males in Iran^11^, adolescent smokers in Korea^12^, and US high school students^13^.

The tobacco industry in the Philippines has been described as the ‘strongest tobacco lobby in Asia’^14,15^. According to the 2021 Global Adult Tobacco Survey in the Philippines, 14.5% of Filipino adults smoked tobacco daily^16^. However, the relationship between age at smoking initiation and smoking intensity has not yet been studied in the country. To address this research gap, this study aims to identify the relationship between age at smoking initiation and smoking intensity among Filipino daily smokers, with the hope to contribute to the understanding of the behavioral and health trajectories of Filipinos who start smoking at an early age. The results of this study can also contribute to the development of policies and programs designed to prevent and reduce tobacco use among Filipinos.

## METHODS

### Data source

Secondary data analyses were performed. Data from the 2021 Global Adult Tobacco Survey (GATS) Philippines were obtained. The 2021 GATS Philippines is a nationwide household survey that aims to monitor tobacco use among adults and track key tobacco control indicators. It adopted the standard protocol for the implementation of GATS, which was implemented by the Philippine Statistics Authority in coordination with the Department of Health of the Philippines. Data collection was from November to December 2021. Full information on the methodology of the 2021 GATS Philippines is available in its final report^16^. The anonymized public use file that the present research used is also published on the PSA website^17^.

### Sampling

The 2021 GATS Philippines implemented a multistage, geographically clustered sample design. The primary sampling unit was the enumeration area, while the secondary sampling unit was the housing unit. There were 20971 households that were sampled. For each household, one individual aged ≥15 years was randomly chosen to respond to the individual questionnaire. There were 18466 completed interviews.

For the current study, individuals identified to be daily smokers based on the question: ‘Do you currently smoke tobacco on a daily basis, less than daily, or not at all?’ (i.e. answered daily), are covered (n=2610). The final dataset comprised 2260 participants after removing those with missing information.

### Variables and measures

Table 1 summarizes the variables included in the study, the corresponding questions from the 2021 GATS Philippines that measure these variables, and the categorical coding. The outcome variable in the study was smoking intensity, which was measured as the number of manufactured cigarettes smoked daily. The main predictor variable was the age of daily smoking initiation, measured as the age when the individual first started smoking daily.

**Table 1:** Variables of the study, question phrasing from the 2021 GATS, and variable coding for the present research

*Variables*	*Questions from 2021 GATS Philippines*	*Coding for the current research*
**Smoking intensity** (dependent variable)	On average, how many of the following products do you currently smoke each day?	Numerical: number of manufactured cigarettes smoked daily
**Age of daily smoking initiation** (predictor variable)	How old were you when you first started smoking tobacco daily?	Numerical
**Age** (years)	What is the month of your date of birth? What is the year of your date of birth? Your age is calculated as (calculated years). Is this correct?	Numerical
**Sex**	Sex was recorded based on observation.	0=Female 1=Male
**Education level**	What is the highest level of education you have completed?	0=Elementary level or below 1=Above elementary level
**Employment**	Which of the following best describes your main work status over the past 12 months?	0=Not employed 1=Employed
**Income**	Please look at this card and let me know which category your monthly income falls under.	0=4999 Pesos and lower 1=higher than 4999 Pesos
**Smoking rules at home**	Which of the following best describe the rules about smoking inside of your home?	0=Smoking not allowed/no rules 1=Smoking allowed
**Belief if tobacco smoking can cause serious illness**	Based on what you know or believe, does smoking tobacco cause serious illness?	0=No 1=Yes

The study also included potential confounders as controls – variables that are associated with both dependent and independent variables. These were chosen based on previous studies on smoking initiation and intensity. Particularly, these were age, sex, education level, employment, income, smoking rules at home, and belief whether tobacco smoking causes serious illnesses. These controls were also obtained from the 2021 GATS Philippines.

### Data analysis

Data were summarized using descriptive statistics. Frequencies and percentages were calculated for categorical variables, while means and standard deviations were computed for numerical variables. Independent sample t-tests were done to identify whether there were significant differences in smoking intensity between categories of categorical variables, all of which were binary. Pearson correlation analyses were performed to identify relationships among numerical variables. Poisson regression was initially performed to model smoking intensity, but due to evidence of overdispersion, negative binomial regression was used instead. In the first step, age at smoking initiation was entered first to ascertain its unadjusted relationship with the smoking intensity. Potential confounders were added in the second step to identify whether the effect of age at daily smoking onset was consistent. The p-value was set at 0.05, and SAS^®^ Studio^18^ was used to analyze data.

## RESULTS

Table 2 summarizes the characteristics of respondents. The majority of the daily smokers were males (89.78%). The average age of the respondents was 41.33 years (SD=14.06), which indicates that the respondents were typically middle-aged. Most respondents had an education level above elementary (71.73%). A significant majority of the respondents were employed (85.13%). Slightly more than half (54.78%) had income >4999 PHP (1000 Philippine Pesos about US$17). In terms of smoking rules at home, nearly 3 in 10 (28.14%) reported living in a home that allowed smoking. A large majority (93.14%) believed that tobacco smoking could cause serious illness. The average smoking intensity was 9.50 cigarettes per day (SD=7.26), while the mean age of daily smoking initiation was 20.93 years (SD=6.35).

**Table 2:** Profile of daily smokers in terms of sex, education level, employment, income, smoking rules at home, belief that tobacco smoking can cause serious illness, age, smoking intensity, and age at smoking initiation, based on the 2021 GATS, a cross-sectional survey (N=2260)*

*Variables*	*n*	*%*
**Sex**		
Male	2029	89.78
Female	231	10.22
**Education level**		
Elementary level or lower	639	28.27
Higher than elementary level	1621	71.73
**Employment**		
Not employed	336	14.87
Employed	1924	85.13
**Income** (PHP)		
≤4999	1022	45.22
>4999	1238	54.78
**Smoking rules at home**		
Not allowed/no rules	1624	71.86
Smoking allowed	636	28.14
**Believes that tobacco smoking can cause serious illness**		
Yes	2105	93.14
No	155	6.86
	***Mean***	***SD***
**Age** (years)	41.33	14.06
**Smoking intensity** (cigarettes/day)	9.50	7.26
**Age of daily smoking initiation** (years)	20.93	6.35

* After eliminating cases with missing, refused, and don’t know responses. PHP: 1000 Philippine Pesos about US$17.

Table 3 presents the differences in the mean smoking intensity between categories of variables and t-test results. T-test indicates that there is a statistically significant difference in smoking intensity between males and females, with males smoking more (mean=9.66, SD=7.25) compared to females [mean=8.07 (SD=7.19), t(2258)=3.16, p=0.0016]. A statistically significant difference in smoking intensity was also found between homes that allow smoking and homes that do not allow smoking or with no rules on smoking. Those who reported living in a home that allowed smoking had a higher smoking intensity (mean=10.92, SD=7.65) compared to those from a home where smoking was not allowed or had no rules [mean=8.94 (SD=7.02), t(1078.1)= -5.67, p<0.0001]. Smoking intensity was also higher among those with relatively lower level of education, employed, earning more, and those who do not believe that tobacco smoking can cause serious illness, but the differences were not statistically significant (p>0.05).

**Table 3:** T-tests on the difference between the averages of smoking intensity between groupings of sex, education level, employment, income, smoking rules at home, and belief that tobacco smoking can cause serious illness, based on the 2021 GATS, a cross-sectional survey (N=2260)*

*Variables*	*Mean (SD)*	*t (df)*	*p*
**Sex**		3.16 (2258)	0.0016
Male	9.66 (7.25)		
Female	8.07 (7.19)		
**Education level**		1.31 (2258)	0.1892
Elementary level or lower	9.81 (7.52)		
Higher than elementary level	9.37 (7.15)		
**Employment**		-1.39 (2258)	0.1645
Not employed	8.99 (7.26)		
Employed	9.58 (7.26)		
**Income** (PHP)		-1.67 (2258)	0.0959
≤4999	9.22 (7.14)		
>4999	9.73 (7.35)		
**Smoking rules at home**		-5.67 (1078.1)	<0.0001
Not allowed/no rules	8.94 (7.02)		
Smoking allowed	10.92 (7.65)		
**Believes that tobacco smoking can cause serious illness**		-0.22 (189.96)	0.8257
Yes	9.49 (7.34)		
No	9.60 (5.98)		

* After eliminating cases with missing, refused, and don’t know responses. PHP: 1000 Philippine Pesos about US$17.

In terms of quantitative variables, correlation analyses indicate there is a positive correlation between current age and age at smoking initiation (r=0.23, p<0.0001), suggesting that older individuals tended to start smoking at a slightly older age. A positive correlation also exists between current age and smoking intensity (r=0.09, p<0.0001); older individuals tend to smoke more. It was also observed that smoking initiation and smoking intensity had an inverse correlation (r= -0.12, p<0.0001), suggesting that individuals who start smoking earlier in life tend to have higher smoking intensity.

Table 4 presents the negative binomial regression results in predicting smoking intensity via age at smoking initiation. In the unadjusted analysis, it was revealed that age at smoking initiation is a significant predictor of smoking intensity. For every additional year in the age at which smoking was initiated, smoking intensity decreased by 1.43% (β= -0.0143; 95% CI: -0.0189 – -0.0099, p<0.0001). After controlling for potential confounders, the relationship between the two variables remained significant (β= -0.0156; 95% CI: -0.0204 – -0.0108, p<0.0001). Specifically, for each additional year in the age at which an individual started smoking, the smoking intensity decreased by approximately 1.55%.

**Table 4:** Negative binomial regression on predicting smoking intensity with age at smoking initiation at Step 1 and after controlling for potential confounders at Step 2, based on the 2021 GATS, a cross-sectional survey (N=2260)*

*Parameter*	*β*	*SE*	*95% CI*		*p*
			*Lower*	*Upper*	
**Unadjusted for other variables**					
Age at smoking initiation	-0.0144	0.0023	-0.0189	-0.0099	<0.0001
**After adjusting for other variables**					
Age at smoking initiation	-0.0156	0.0024	-0.0204	-0.0108	<0.0001
Sex (female)	-0.1146	0.0516	-0.2157	-0.0136	0.0262
Age (years)	0.0072	0.0011	0.0050	0.0094	<0.0001
Education level (higher than elementary level)	0.0189	0.0341	-0.0480	0.0857	0.5802
Employment (employed)	0.0683	0.0447	-0.0193	0.1559	0.1267
Income (>4999 PHP)	0.0414	0.0325	-0.0223	0.1051	0.2024
Believes that tobacco smoking can cause serious illness (no)	-0.0064	0.0575	-0.1191	0.1063	0.9111
Smoking rules at home (smoking allowed)	0.1807	0.0322	0.1175	0.2439	<0.0001

Step 1: Smoking intensity was regressed on age at smoking initiation. Step 2: Smoking intensity was regressed on age at smoking initiation while controlling for other variables. Reference values of categorical predictors: males; elementary education and below; not employed; ≤4999 PHP; yes; smoking not allowed/no rules.

* After eliminating cases with missing, refused, and don’t know responses. PHP: 1000 Philippine Pesos about US$17.

Additionally, current age was positively associated with smoking intensity (β=0.0072; 95% CI: 0.0050–0.0094, p<0.0001). For each additional year of age, smoking intensity increased by 0.72%. Females were also less likely to be heavier smokers (β= -0.1146; 95% CI: -0.2157 – -0.0136, p=0.0262). Finally, living in a home where smoking was allowed was significantly associated with higher smoking intensity (β=0.1807; 95% CI: 0.1175–0.2439, p< 0.0001). Individuals living in a home where smoking was permitted had a smoking intensity that was approximately 19.81% higher compared to those living in a home where smoking was not allowed or where there were no rules. No significant effects were found for education level, employment, income, and belief variables (p>0.05).

## DISCUSSION

The study aims to identify the relationship between age at smoking initiation and smoking intensity. Doing so is crucial in contributing to the understanding of the health consequences of smoking in the future as well as future trajectories of smoking behavior. It can also help in the development of effective strategies that aim to prevent and reduce tobacco use. The positive relationship between the two smoking behaviors has been recognized by many authors from different countries for different groups^9-13^. However, such an association has not yet been analyzed in the Philippines. The results of the present research indicated that individuals who began daily smoking at a younger age are more likely to have greater smoking intensity at subsequent ages. Even after controlling for potential confounding variables, the relationship holds. Additionally, the study found that males, older smokers, and smokers living in a home where smoking is permitted had greater smoking intensity as well.

The study agrees with most similar studies that corroborate the view that individuals who start smoking earlier tend to smoke heavier at subsequent ages^9-13^. Data from this research suggest that smoking prevention efforts could target young initiators to prevent diseases and mortality attributable to heavy smoking once they reach older ages. There are strategies that can be effective against smoking initiation. These include the provision of anti-smoking messages, implementation of smoke-free policies, smoking curricula in schools, and restricting adolescents’ ability to buy cigarettes, among others^19^. Even though the commencement of daily smoking is the regressor of interest in this study, the present study also found other interesting results that deserve recognition.

The results of this study also indicated that being male is associated with a greater number of cigarettes smoked per day. This is in congruence with recent literature among adult smokers in Tehran^20^, urban emergency department patients from Northern California^21^, and adolescent smokers in Canada^22^. It can be argued that the sociocultural environments of men and women explain the gender differences in smoking^23^. In the Philippines, parents are more likely to approve of their sons’ drinking and smoking activities compared to their daughters, which highlights the difference in the acceptability of risk-taking between men and women^24^.

The nexus between current age and smoking intensity has also been documented by literature. The current research findings indicate that older people tend to smoke more. The findings are consistent with previous studies. In a cohort study in west Iran, those aged 56–65 years showed the highest adjusted incidence risk ratio for smoking intensity compared to those in the age groups of 46–55 and 35–45 years^25^. Also, a Canadian study has also illustrated that cigarettes per day among daily smokers were highest among older adults (aged ≥30 years) followed by young adults (aged 18–29 years) and youth (aged 15–17 years)^26^. One possible explanation could be that older individuals may be more experienced with tobacco use and may have a longer history of smoking^20^. Additionally, these might be formed habits that are difficult to stop^27^.

Anti-smoking rules at home can serve as anti-smoking socialization among adolescents and adults^28^. In the current study, smoking rules at home also predicted smoking intensity, where individuals living in a home that allow smoking are more likely to be heavier smokers. Similarly, a study comprising multi-year GATS data from low-income countries found out that the average number of cigarettes per day is highest among smokers living in a home where smoking was allowed compared to those living in partial and complete smoke-free homes^29^. The present finding suggests that promoting smoke-free homes may reduce smoking intensity among daily smokers. Banning smoking at home not only reduces the smoking intensity but is also associated with other anti-smoking behaviors such as successful quitting – a relationship observed in the US population^30^.

### Limitations

While the study yielded interesting insights, this study has limitations. First, results should not be interpreted in a causal manner since only one round of GATS data was used, and the GATS design is cross-sectional. Second, the use of secondary data limited the ability to explore other variables that could be included in the list of potential confounders. For instance, alcohol use was found to be associated with both smoking initiation^31^ and intensity^32^. It should also be noted that electronic cigarettes, which have become popular and accessible among youth in South-East Asia^33^, are not covered by the present study. Finally, GATS data collection relied on face-to-face self-reports; hence, false information and social desirability bias could have been present.

## CONCLUSIONS

The study provides evidence that higher smoking intensity may be linked to early smoking initiation among Filipino adults who smoke daily. The results may support interventions that target younger ages. Further studies on smoking intensity among Filipinos are warranted, particularly by adding more relevant variables not covered by this research.

## Data Availability

The data supporting this research are available from the author on reasonable request.
